# Intermediate, but not average: The unusual lives of the nuclear lamin proteins

**DOI:** 10.1016/j.ceb.2023.102220

**Published:** 2023-08-22

**Authors:** Abigail Buchwalter

**Affiliations:** 1 Cardiovascular Research Institute and Department of Physiology, University of California, San Francisco, San Francisco, CA, USA; 2 Chan Zuckerberg Biohub, San Francisco, CA, USA

## Abstract

The nuclear lamins are polymeric intermediate filament proteins that scaffold the nucleus and organize the genome in nearly all eukaryotic cells. This review focuses on the dynamic regulation of lamin filaments through their biogenesis, assembly, disassembly, and degradation. The lamins are unusually long-lived proteins under homeostatic conditions, but their turnover can be induced in select contexts that are highlighted in this review. Finally, we discuss recent investigations into the influence of laminopathy-linked mutations on the assembly, folding, and stability of the nuclear lamins.

## Introduction: the nuclear lamin proteins

The lamins are the most ancient and founding members of the intermediate filament (IF) family of cytoskeletal proteins and are enriched within the cell nucleus. IFs are classified into six subtypes based on sequence similarity; the lamins are type V IFs. While the lamins were long thought to be the only IF family members found within the nucleus while all other IF subtypes were presumed to be restricted to the cytoplasm, recent work has challenged this assumption. In fact, IF proteins including keratins (which are type I and II IFs) and nestin (which is a type VI IF) harbor nuclear localization sequences (NLSs) and can enrich in the nucleus under some conditions, where they influence nuclear morphology and the enrichment of other nuclear proteins [1,2].

While the functions of the lamins are most well understood in metazoans, prototypical lamins have also been described in amoebozoa [3]. The mammalian lamins will be the main focus of this review. In mammals, three lamin genes encode four major lamin protein isoforms (lamins A and C from *LMNA*; lamin B1 from *LMNB1;* and lamin B2 from *LMNB2*). Knockouts of each of these genes are lethal at or shortly after birth in mice [4–6], indicating the non-redundant and essential functions of each of these proteins.

The lamins assemble into the nuclear lamina, a whisper-thin interface between the nuclear envelope (NE) membrane and the chromatin mass that is tens of nanometers thick. This unique interface enriches hundreds of proteins from the nucleoplasm and NE membrane via lamin-binding interactions to enable two essential cellular functions. Firstly, the lamina provides structural rigidity to the nucleus and connects the nucleus to the cytoskeleton via the linker of nucleoskeleton and cytoskeleton (LINC) complex, a multiprotein complex that spans the NE and binds directly to both the lamina and the cytoskeleton [7]. Through these connections, the lamina helps the nucleus to sense and adapt to force and to orient during cell migration (see reference [7] for an in-depth review). The second major function that the lamina performs is a genome regulatory one: it tethers large tracts of heterochromatin to the nuclear periphery. These so-called “lamina-associated domains” of heterochromatin contribute to the stable silencing of gene-poor repetitive heterochromatin, thus ensuring genome integrity, and promote the cell-type-specific repression of select genes, thus sculpting cell fate (see reference [8] for a detailed review). This review will discuss how dynamic regulation of the lamin proteins enables and modulates these functions of the lamina.

### Lamin biogenesis and assembly

**Figure 1:**
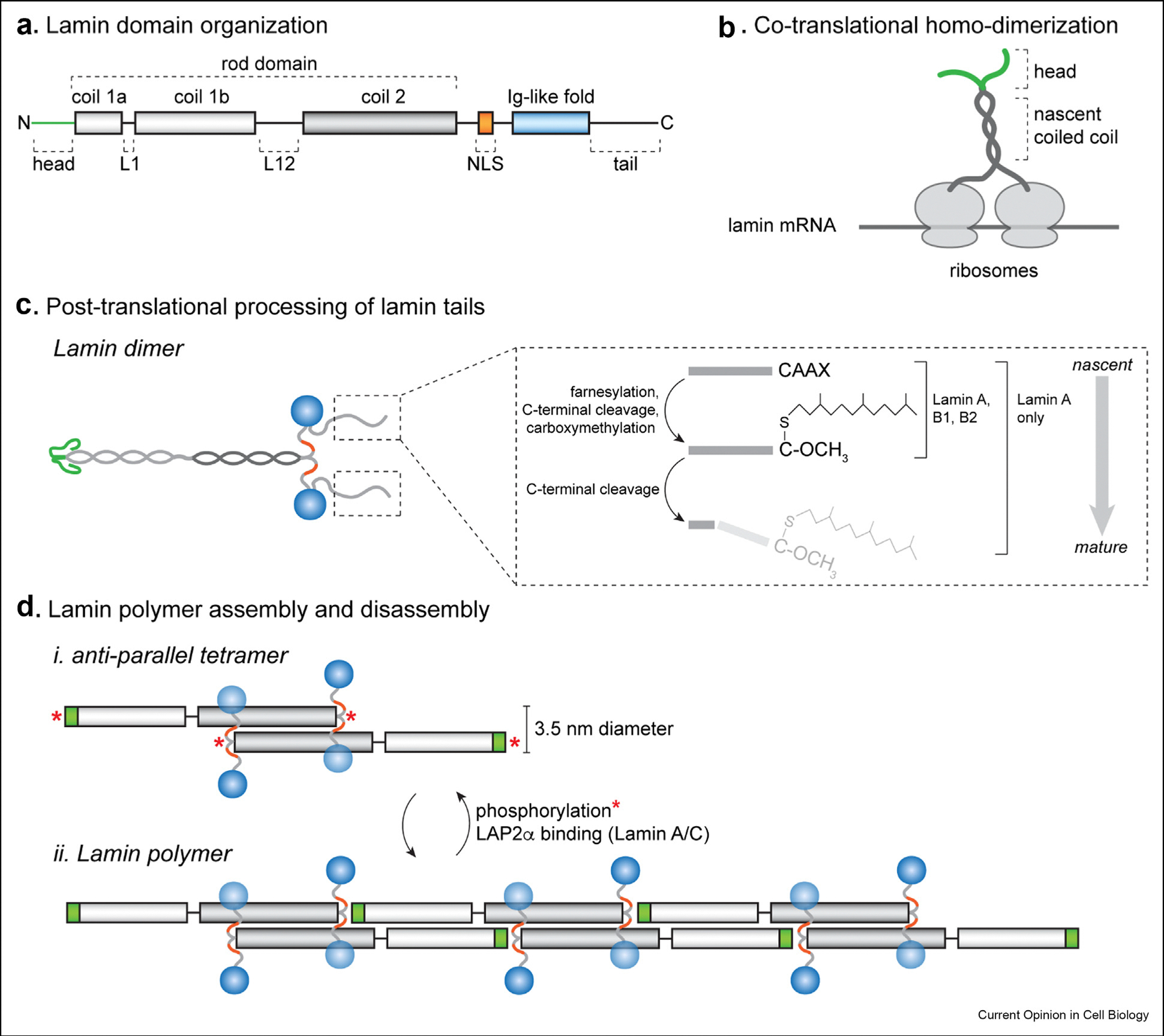
Domain organization and assembly of the nuclear lamin proteins. (**a**) All lamin family members share a conserved domain organization of an unstructured N-terminal head domain (green), a rod domain composed of a-helical coils separated by two unstructured linkers (L1 and L12); a nuclear localization sequence (NLS); an immunoglobulin-like (lg-like) fold; and an unstructured tail. (**b**) Nascent lamin proteins homo-dimerize co-trans-lationally via their coiled coil domains as their nascent chains emerge from nascent ribosomes (see ref [14]). (**c**) Lamins (with the exception of the lamin C isoform) undergo processing of their C-terminal tails after translation and dimerization. Lamin A, B1, and B2 contain a C-terminal CAAX box motif that is farnesylated, cleaved after the modified cysteine, then carboxy-methylated. Lamin B1 and B2 are permanently farnesylated, while lamin A’s lipid-modified tail is cleaved off by the Zmpste24 protease. (**d**) Lamin assembly proceeds by formation of anti-parallel dimers followed by head-to-tail polymerization mediated by electrostatic interactions between the N-terminal head domain (green) and the C-terminal end of the rod domain. The globular lg fold domain is connected to the filament by a flexible linker and can thus freely move about the filament axis (blue). Note that lamin polymers have no overall polarity. Lamin polymers can be disassembled by phosphorylation at head and tail domain sites (*). Lamin A/C polymerization is antagonized by LAP2a binding.

While no complete atomic-resolution structure of a lamin protein or polymer has been solved, structural biologists have pieced together many elements of lamin filament assembly and organization (reviewed recently in [9]) (Figure 1). Lamins share a conserved domain organization of an N-terminal head domain, two α-helical coiled-coil-forming domains separated by short flexible linkers, an NLS, a globular immunoglobulin-like (Ig) β fold domain, and an unstructured C-terminal tail [9] (Figure 1a). Lamin monomers dimerize via their α-helical domains to form a central coiled-coil; these dimers form anti-parallel tetramers [10], which can assemble into filaments via head-to-tail polymerization of the coiled-coil domains (Figure 1c and d). The globular Ig-like β fold domains hang off the main axis of the filament. Within the cellular environment, endogenous lamin filaments are visible as fibers ~3.5 nm (nm) in diameter, which are consistent with a coiled-coil tetramer [11]. While the lamins have the capacity to densely bundle into higher-order “para-crystalline arrays” when purified to homogeneity *in vitro* [12] or when overexpressed in cell culture [13], it is unclear to what extent polymer bundling occurs within the cellular milieu, or whether this bundling is reversible or regulated.

The avid polymerization capacity of the lamin proteins creates a unique challenge for their biogenesis: polymerization must be kept in check during synthesis in the cytosol and must only be unleashed once these proteins reach the nucleus. Lamins dimerize co-translationally via their coiled-coil domains as they emerge from the exit tunnel of neighboring ribosomes that are engaged with the same transcript [14**] (Figure 1b). This elegant mechanism ensures the complete fidelity of homodimerization and precludes heterodimerization even between closely related lamin isoforms. As dimerization begins before the proteins are even fully translated, this discovery establishes dimerization as the very first processing event that the lamins undergo, even preceding extensively studied enzymatic modifications of their C-termini (reviewed in [15]). Briefly, lamin A, lamin B1, and lamin B2 are farnesylated on their C-termini by farnesyltransferase. Lamin B1 and lamin B2 retain their farnesylated tails, while lamin A’s farnesylated tail is removed by the ZMPSTE24 protease (Figure 1c). Lamin C, a shorter isoform encoded by the *LMNA* gene, is spared from these modification steps. The lamins are imported into the nucleus via NLS-mediated import; when the lamin NLS is engaged by importin-α, lamin polymerization is inhibited [16]. This observation suggests that importin-α could perform a chaperone-like function for lamin subunits in the cytosol, and that inhibition of polymerization would be relieved only in the nucleus where abundant RanGTP can induce the dissociation of importin-α/β heterodimers from the lamin NLS.

### Features of lamin polymers

Lamin polymers are highly flexible, with a short persistence length of ~200 nm [11,17], and can extend or compress along the filament axis by lateral sliding of adjacent coiled-coils along each other, enabled by flexible linker domains between the coils [18]. These dynamic, flexible polymers can resist incredibly high forces by strain-stiffening, where coiled-coil segments undergo an α-helical to β-sheet transition under high strain [19]. A-type and B-type lamin polymers contribute distinct biophysical features to the lamina. Lamin A/C polymers appear to be especially mechanoresponsive and can alter their folding and/or polymerization in response to mechanical stimuli such as substrate stiffness, cell spreading, or cell compression [20–22]. Some of these conformational changes have been inferred from changes to antibody epitope accessibility. For instance, epitopes corresponding to the N terminus, C terminus, or Ig fold of lamin A/C are more accessible on the apical surface than on the basal surface of the nucleus within adherent cells grown in a monolayer [20,23]. An Ig fold epitope is more accessible throughout the nucleus when adherent cells can spread out in low-density conditions than when cells are compressed in high-density culture conditions [21]. Conformational changes in the lamin A/C Ig fold have also been detected by changes to the accessibility of an endogenous cysteine residue within the Ig fold, Cys 522, to fluorescent labeling in response to shear stress [24]. Altogether, these studies indicate that lamin A/C is an especially conformationally dynamic polymer.

While cytosolic IF polymers undergo subunit exchange, it is unclear whether this occurs within lamin polymers. Time-lapse fluorescence microscopy of cytosolic IFs has revealed severing and reannealing of polymers, as well as elongation by end-to-end annealing [25–27]. All IFs are present both in mature filament and soluble oligomeric precursor pools; in the case of vimentin (a type III IF), the latter pool has been shown to exchange individual subunits in a rapid and ATP-dependent manner [28], while mature filaments rarely undergo individual subunit exchange, suggesting that IF polymers increase in stability as their assembly progresses [27]. It is unclear how generalizable these observations are across IF subfamilies, and it is relevant to note that the lamins form slender ~3.5 nm tetrameric filaments [11] while cytosolic IFs typically form more stout ~10 nm fibers [11,27]. Similar explorations into lamin filament dynamics have not yet been reported but would be impactful for determining similarities and differences between nuclear lamin polymers and their distant cytoplasmic relatives.

While many questions about lamin filament dynamics remain, cofactor proteins and post-translational modifications (PTMs) have been shown to modulate lamin assembly state.

### Control of lamin polymerization

Lamins are present both as mature polymers that are enriched at the nuclear periphery and as a soluble pool within the nucleoplasm. In the case of lamin A/C, one cofactor protein has been implicated in influencing the balance between these distinct assembly states: lamina-associated polypeptide 2α (LAP2α). LAP2α limits the polymerization of purified lamin A/C *in vitro* and increases the solubility and mobility of the lamin A/C proteins within the nucleoplasm, without altering their phosphorylation state [29]. In the presence of LAP2α, lamin A/C can interact with transcriptionally active genes within the nucleoplasm [30]. In the absence of LAP2α, lamin A/C in the nuclear interior forms a more insoluble and perhaps more extensively polymerized meshwork that is poorly detected by an antibody that recognizes lamin A/C’s N-terminus [29,31]. This same antibody detects lamin A/C at the nuclear lamina in the absence and presence of LAP2α, implying differences in lamin A/C assembly and/or protein-protein interactions in the nucleoplasm *versus* the nuclear periphery that are modulated by LAP2α.

The lamina meshwork must be completely disassembled at the onset of mitosis to give the mitotic spindle access to the genome. Lamina disassembly is achieved by phosphorylation of sites flanking the central coiled coil by Cdk1 and other kinases; these phospho-modifications disrupt head-to-tail interactions along the filament axis [32] (Figure 1d). For example, human lamin A/C is phosphorylated at sites including serine 22 (Ser 22) and serine 392 (Ser 392) in mitotic cells [33]. Phosphorylation of the N-terminal head domain appears to be especially potent at disrupting lamin filaments, as mutating these sites to prevent phosphorylation is sufficient to prevent depolymerization of lamin A [33], while phosphomimetic substitutions at either Ser 22 or Ser 392 displace lamin A from the lamina and into the nucleoplasm [34]. Recent work has sketched out a model to explain how N-terminal phosphorylation drives lamin polymer disassembly: head-to-tail assembly is stabilized by inter-subunit electrostatic interactions between the N-terminal head domain and the C-terminal end of the coiled-coil domain [18]. Phosphorylation of N-termini at sites including Thr 19 and Ser 22 introduces negative charge that may electrostatically interact with positively charged residues in the head domain of the same lamin monomer. This interaction would replace an intermolecular interaction with an intra-molecular one, thus allowing polymers to disassemble [32].

Intracellular pathogens co-opt lamin phosphorylation to disrupt the nucleus; for instance, herpesviruses replicate within the nucleus and bud across the NE and express kinases that phosphorylate and disassemble the lamina [35] to increase access of nascent virions to the NE. Immune cells dismantle their own lamina in order to respond to extracellular pathogens. When activated by the presence of a nearby pathogen, neutrophils self-destruct, releasing their chromatin as neutrophil extracellular traps (NETs) that ensnare pathogens. Lamin B1 phosphorylation by protein kinase C (PKC) appears to promote nuclear rupture and enable chromatin extrusion in activated neutrophils [36].

While high occupancy of lamin phospho-sites completely disassembles the lamina [37], lower levels of phosphorylation may modulate lamin assembly state and function while maintaining nuclear integrity in non-mitotic cells [34]. For instance, phosphorylation of lamin A/C at Ser 22 allows this protein to take on distinct functions within the nucleoplasm of interphase cells, where it interacts with enhancers of actively transcribed genes – in contrast to the association of repressed genes with non-phosphorylated lamins at the nuclear periphery [38]. This phospho-modification is also induced as an acute response to heat shock, perhaps mediating adaptive changes to nuclear structure and/or gene expression [39]. Lamin A/C Ser 22 phosphorylation is mechanoresponsive and interconnected with nuclear rounding; this phospho-modification rapidly increases when adherent mesenchymal cells are grown in suspension and conversely, rapidly decreases as cells adhere and spread on a substrate [40]. This relationship is also observed in epithelial cell types, where lamin A/C phosphorylation increases as cells become compressed within an epithelial monolayer [22]. Altogether, these studies indicate that control of lamin polymerization both enables novel functions of lamin A/C within the nucleoplasm, with consequences on gene expression, and modulates the biophysical properties of the nuclear lamina, with consequences on nuclear shape and stiffness.

Lamin polymer disassembly by these or other mechanisms is likely to be a prerequisite for degradation of individual Lamin subunits, which appears to occur very rarely in most contexts.

### Lamins are extremely long-lived proteins

**Figure 2:**
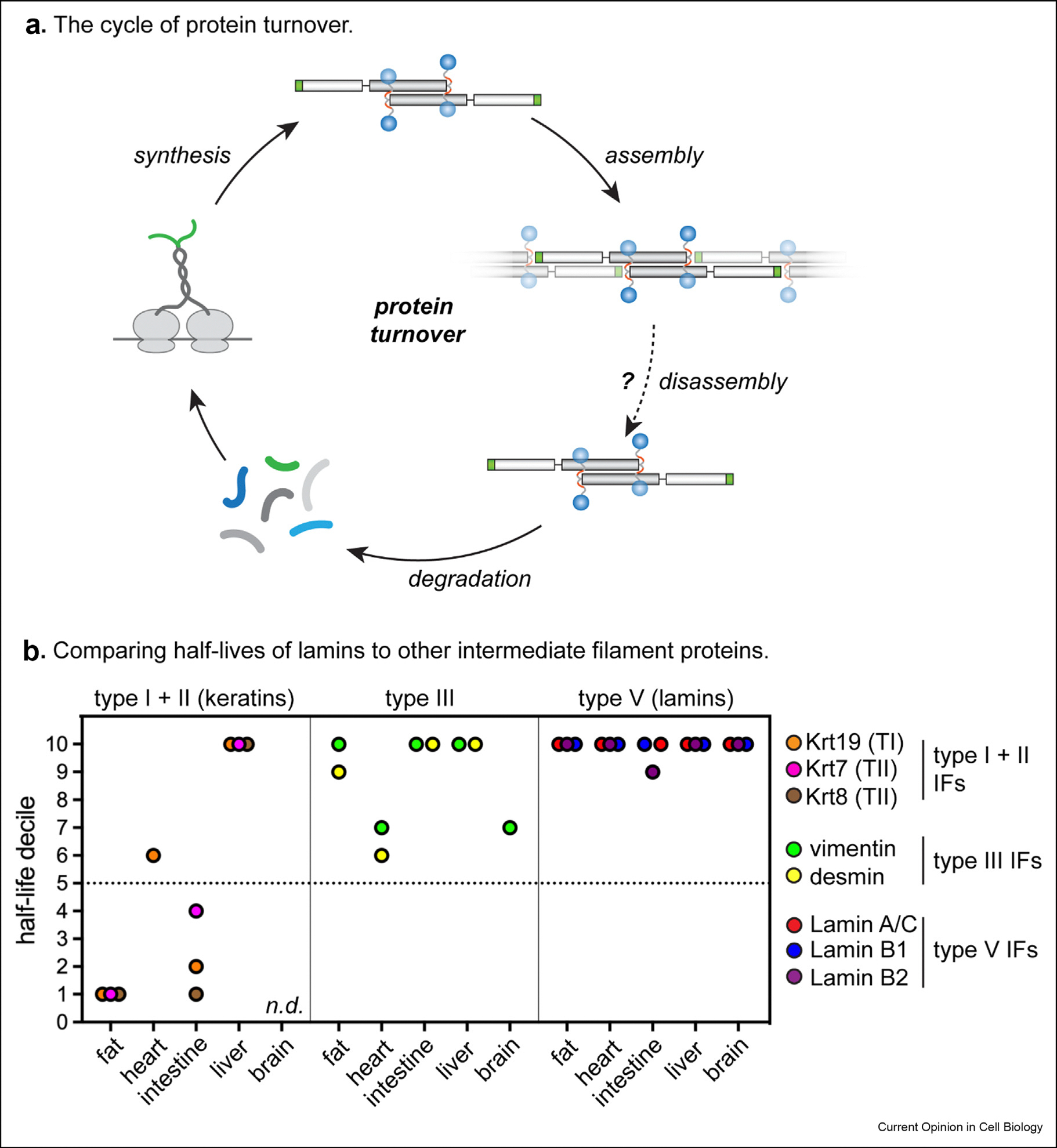
(**a**) The rate of protein turnover depends on the rates of protein synthesis and protein degradation. (?) indicates that it is unknown whether Jamin polymer disassembly precedes protein degradation. (**b**) The Jamin proteins are extremely long-lived proteins with very slow turnover rates compared to most other proteins and compared to other intermediate filament family members. Lamin isoforms generally have a half-life in the top 10% (10^th^ decile) in the proteomes of the fat, heart, intestine, liver, and brain of healthy young adult mice. Rep-resentative Type I, II, and Ill IF family members have shorter and more variable half-lives in the same tissues. Fat, heart, intestine, and liver data re-analyzed from [41]; brain data re-analyzed from [82]

Individual proteins undergo degradation at distinct rates that are influenced by intrinsic sequence features, three-dimensional structure features, and environmental factors [41]. When compared to the half-lives of most proteins and other IF family members within mammalian tissues, the lamins are extremely long-lived: their lifetimes are generally within the top 10% (Figure 2). Because average protein turnover flux varies from tissue to tissue, the absolute lifetimes of the lamin proteins do as well. At their longest-lived extreme, lamin B1 and lamin B2 have half-lives of approximately 4 months and 2 months in the mouse brain, respectively (where they are each highly expressed), while lamin A/C has a half-life of approximately 1 month in the mouse heart (where it is highly expressed) (Table 1).

The extremely long lifetimes of the lamin proteins may imply that lamin polymers are extremely stable and infrequently undergo subunit exchange, as disassembled subunits are likely to be a better substrate for protein degradation machineries. A similar rationale has been proposed to explain the long lifetimes of nuclear pore complex subunits and of replication-dependent histones, which are in multiprotein complexes that may never disassemble throughout a cell’s lifetime [42,43]. While the mechanism of lamin protein turnover is unknown, other IF family members such as desmin (a cytosolic type III IF) must be disassembled from polymers by phosphorylation, ATPase activity, and ubiquitination before targeting to the proteasome during muscle remodeling [44]. Compared to most IFs, keratins undergo unusually rapid assembly/disassembly cycles [27], have been shown to be degraded rapidly in response to various cellular signals [45,46], and have shorter half-lives in many tissues (Figure 2). These correlations suggest that IF polymer disassembly enables proteolytic degradation of IF subunits.

The absence or presence of specific PTMs may also influence lamin stability. The lamins are subject to a wide range of PTMs; while a subset are highlighted here, lamin PTMs have been comprehensively reviewed elsewhere [47]. For instance, phosphorylation-mediated lamin polymer disassembly could make subunits more accessible to degradation [24], while the diminution of this modification in non-dividing cells might be sufficient to protect the lamins from degradation. Alternatively, it is possible that additional PTMs actively protect the lamins from degradation. One particularly interesting lamin modification that has been recently described is lysine acetylation, which is deposited on numerous lysines along the lamins’ α-helical coiled coil domains by the MOF acetyltransferase [48]. Disruption of lamin acetylation causes disorganization of the lamina, increased lamin solubility, and nuclear softening [48], indicating that acetylation influences the biochemical properties of the lamina. It is tempting to draw a parallel between lamin acetylation and tubulin acetylation, which increases microtubule perdurance by altering tubulin subunit packing in a way that makes microtubules more resilient to strain [49]. It is unclear whether acetylation could similarly alter the packing of lamin subunits or resilience of lamin polymers. Alternatively, recent evidence indicates that lysine acetylation may protect proteins from degradation by competitively preventing the conjugation of ubiquitin to the same lysine [50], but whether such a competitive mechanism enhances lamin stability is unknown. The lamins are readily detected in proteomic surveys of ubiquitin-modified proteins [51], but when and whether ubiquitination induces lamin degradation remains unclear. It was recently reported that the ubiquitin paralog UBBP4 is expressed in most mammalian tissues and is conjugated to many proteins including the lamins [52]. Interestingly, UBBP4 cannot engage with the proteasome [52], raising the possibility that this modification could also competitively inhibit lamin poly-ubiquitination and proteasome engagement.

### Consequences of the long lifetime of the lamin proteins

Long protein lifetime confers the ability to perform a function over a long timescale. When considered in this light, the long lifetime of the lamins may endow these proteins with the ability to act as extremely stable scaffolds of nuclear organization. However, long protein lifetime may also be a weakness. Long-lived proteins are vulnerable to molecular aging, which occurs at the level of individual protein molecules and includes covalent modifications, misfolding, and irreversible aggregation [43]. In some cases, molecular aging of long-lived proteins has been directly linked to age-linked tissue dysfunction. For example, the crystallins are extremely long-lived proteins within the eye lens that irreversibly aggregate over time and cause cataracts in aged individuals [43]. Recent evidence indicates that the lamins may also undergo age-linked aggregation. Lamin A/C aggregates in the heart during physiological aging in the killifish, a short-lived fish that is an emerging model for vertebrate aging [53].

Long-lived proteins are uniquely resistant to genetic perturbations because they are so rarely synthesized and degraded. In practical terms, this can manifest in the poor potency of inducible genetic knock-out or transcript knock-down approaches, especially in the context of adult tissues. For instance, ablation of the *Lmnb1* gene by *Cre* recombinase in the postnatal mouse retina cannot effectively displace the lamin B1 protein from terminally differentiated rod photoreceptor cells, due to the protein’s extremely long lifetime in that cell type [54]. The persistence of long-lived proteins after the gene that encodes them has been inactivated may also introduce latency in the response to gene therapies. Recent evidence suggests that the long lifetime of lamin A/C may slow response to gene therapies for Hutchinson-Gilford progeria syndrome (HGPS). An antisense oligonucleotide-based gene therapy cannot effectively clear the toxic Progerin protein from the heart of progeroid mice even after five months of treatment, long after the RNA has been repressed to nearly undetectable levels [55].

### Induction of lamin protein turnover

While the lamin proteins have extremely long lifetimes under homeostatic conditions, there are select contexts where their turnover has been observed, often concomitant with morphological changes to the nucleus and altered cellular behaviors. Generally, lamin-mediated turnover has been linked to protease activity or to induction of macroautophagy. The lamina is a target of caspase proteases during apoptosis (reviewed in [56]). Recently, selective caspase-mediated proteolysis of lamin A/C has been implicated in nuclear remodeling in macrophages responding to a pro-inflammatory stimulus [57].

Autophagy-mediated lamin turnover has been described during cellular senescence [58]. Senescence is an irreversible cell-cycle arrest that is associated with increased autophagic flux and lysosomal biogenesis which enable widespread cellular remodeling [59]. Downregulation of the lamin proteins occurs when cells enter senescence in response to stressors including telomere shortening (replicative senescence), acute DNA damage, or oncogene expression [58–60]. While loss of lamin A, lamin B1, and lamin B2 in senescent cells has been observed, lamin C appears to be unaffected by senescence [59,60]. lamin A/C or lamin B1 depletion accelerates senescence onset [59], while overexpression of lamin B1 sustains higher levels of proliferation in cells nearing replicative senescence [61], indicating that lamin depletion promotes senescence entry. Degradation of the lamins during senescence induction requires autophagy; at least for lamin B1, interaction with the autophagy adaptor protein LC3 promotes its autophagy-mediated degradation [58,59].

Autophagy-mediated lamina turnover can also be induced by intracellular pathogens. Herpes simplex virus (HSV) replicates within the host cell nucleus and forms virions by budding across the NE. To accomplish this, viral kinases phosphorylate and locally disassemble the lamina [35]. In addition, autophagy-mediated degradation of the lamina promotes HSV egress specifically within immature dendritic cells (DCs) [62]. DCs detect and present antigens to lymphocytes and are thus key mediators of immune responses to pathogens. However, immature DCs can become infected by HSV and co-opted to produce large numbers of infectious virions, which is enabled by autophagy-mediated degradation of all lamin isoforms to facilitate virion budding. Interestingly, HSV-infected mature DCs do not induce lamin autophagy as effectively and produce far fewer infectious virions [62].

Several features of lamin autophagy remain unclear and should be revisited in light of recent advances in our understanding of the mechanisms of selective macroautophagy. For instance, autophagy may be directed by ubiquitination, occur independently of ubiquitination, or be mediated by other PTMs [63]; protein clients can be selected for autophagy either as individual protein subunits or as part of large aggregates [64]; and a growing list of substrate-specific autophagy receptor proteins promote selective autophagy by mediating engagement of client proteins with LC3 [65]. The modifications and interactions that promote lamin autophagy remain comparatively unknown. Further, how these nuclear proteins come to be enclosed within autophagosomes and digested within cytosolic lysosomes is unclear. Autophagy clients characteristically become enclosed within an LC3-decorated isolation membrane that becomes an autophagosome, which eventually fuses with a degradative lysosome within the cytoplasm. Are the lamins engaged directly by autophagy adaptor proteins from the nucleoplasm, and if so, how are they extracted from the nucleus for lysosomal delivery? Alternatively, does the nuclear periphery – including the membranes and proteins of the NE along with the lamins – bud toward the cytosol in order to rendezvous with a lysosome?

Lamin ubiquitination may be a key step toward initiating turnover by either the proteasome or by autophagy. Protein substrates are selected for ubiquitination by specific E3 ubiquitin ligases, and a small number of E3 ubiquitin ligases have been implicated in lamin ubiquitination and/or lamin turnover. One E3 ligase of interest is the tumor suppressor SMURF2 [66]. SMURF2 interacts with lamin A/C and can catalyze its poly-ubiquitination. Over-expression of SMURF2 decreases the steady-state levels of lamin A/C, while knockout of this enzyme has the opposite effect [67], implying that SMURF2 may play a major role in controlling the degradation of lamin A/C. Inhibition of autophagy, but not the proteasome, blocks SMURF2’s effects on lamin A/C, suggesting that SMURF2 promotes autophagy-mediated degradation. The E3 ubiquitin ligases Siah1, RNF123, and HECW2 have also been implicated in lamin ubiquitination, but it is less clear how large an influence these enzymes exert on lamin A/C protein turnover rate and steady-state protein abundance [68–70]. Generally, the physiological contexts where lamin ubiquitination may be induced by these or other ubiquitin ligases remain unclear. Further, it is unknown whether these or other ubiquitin ligases attach monomeric ubiquitin and/or poly-ubiquitin chains to the lamins, which are decoded differently by cellular ubiquitin-interacting machineries to direct the fate of ubiquitinated proteins (reviewed in [71]).

Recent work has uncovered a potential new axis of lamin protein regulation via cross-talk with other IFs. One noteworthy example is the type VI IF nestin, which is highly expressed in many stem cells, progenitor cells, and cancer cells and can access the nucleus via an NLS. In glioblastoma cells, nestin interacts with lamin A/C [1]; when its expression is inhibited, lamin A/C protein levels rapidly diminish by a mechanism that involves poly-ubiquitination and proteasome activity [1,72]. However, it remains to be determined whether nestin influences lamin A/C stability in other cell types. If nestin loss is sufficient to destabilize lamin A/C, one would predict that the expression of these two related IFs would be tightly correlated across tissues and cell types. This is not the case; nestin is highly expressed in many pluripotent lineages, while lamin A/C is abundant in many differentiated tissues. It therefore seems more probable that additional factor(s) mediate lamin A/C turnover in response to nestin depletion in glioblastomas and possibly in other cell types.

### Effects of laminopathy mutations on lamin proteostasis

A wide range of mutations to the *LMNA* gene, as well as a more limited set of mutations to the *LMNB1* and *LMNB2* genes, cause a phenotypically diverse group of “laminopathy” syndromes [73]. As of this writing, over 300 missense, frameshift, nonsense, and splice site mutations throughout the *LMNA* sequence have been reported in the NIH ClinVar database as pathogenic drivers of cardiomyopathy, muscular dystrophy, lipodystrophy, neuropathy, and other rarer syndromes. It is likely that the molecular consequences of these numerous mutations are just as phenotypically diverse as the diseases that they cause, but only a small subset of these mutations have been studied in detail. Some laminopathy mutations appear to destabilize the lamin A/C proteins, such as the cardiomyopathy-linked S143P and ΔK32 mutations, which undergo increased poly-ubiquitination and accelerated turnover [74,75]. On the other hand, some mutations appear to induce lamin A/C aggregation and/or protein accumulation due to impaired turnover. One example may be the autosomal dominant splice site mutation (G608G) that generates the “progerin” protein isoform which causes the rare and devastating Hutchinson-Gilford progeria syndrome (HGPS). The progerin mutant retains the farnesylated tail that is normally cleaved from pre-lamin A during post-translational processing (see Figure 1c). Progerin thickens and distorts the nuclear lamina [76] and appears to sequester and dominantly interfere with the function of other nuclear proteins [77]. We recently used metabolic labeling in HGPS mice to learn that progerin exhibits impaired protein turnover and accumulates over time in the diseased heart, but not in other tissues [78]. Altered lamin assembly and/or increased aggregation propensity may be a general property of laminopathy mutations. In a recent study, 178 lamin A/C missense mutations were expressed as GFP fusion proteins in cultured skeletal muscle and cardiomyocytes; many of these mutations caused lamin A/C mislocalization into intranuclear aggregates and decreased protein solubility [79]. It is possible that laminopathy mutations over-burden and eventually disrupt the function of the proteostasis machinery, as lamin A/C mutations including S143P, ΔK32, and G608G are associated with decreased proteasome activity [74,75,78,80], while patient cells harboring an E262K atypical progeria mutation accumulate ubiquitinated and misfolded proteins [81]. These observations suggest that proteostasis-correcting interventions such as induced degradation of toxic proteins or guiding protein refolding with molecular chaperones could be effective therapeutic approaches for laminopathy diseases. One way to make progress on such approaches would be to define and then co-opt the cellular pathways that control lamin protein folding, polymer disassembly, or protein degradation.

## Conclusion

Lamin folding, polymerization, and turnover rate are dynamically responsive to signals from the cellular environment ranging from post-translational modifications to mechanical stimuli. We are just beginning to define the nature of these dynamic changes to lamin polymers and to explore how these changes are transduced into context-specific cellular functions. By understanding how the lamin proteins interpret and adapt to cellular cues, we may be able to decipher the variable consequences of laminopathy disease mutations.

## Figures and Tables

**Table 1:** Half-lives of Lamin proteins in mouse tissues.

Tissue	Lamin A/C	Lamin B1	Lamin B2	Proteome median	Reference

Fat (white adipose)	17 days	60 days	53 days	6 days	Hasper et al., Mol Syst Biol 2023
Heart	22 days	86 days	83 days	6 days	Hasper et al., Mol Syst Biol 2023
Intestine	5 days	5 days	3.5 days	2 days	Hasper et al., Mol Syst Biol 2023
Liver	9 days	15 days	10 days	2 days	Hasper et al., Mol Syst Biol 2023
Brain (cerebellum)	35 days	123 days	70 days	8 days	Fornasiero et al., Nat Comms 2018

## Data Availability

No data was used for the research described in the article.
